# Perisaccadic perception of flashing stimulus: Spatial and temporal aspects

**DOI:** 10.1177/03010066261421536

**Published:** 2026-03-18

**Authors:** Rytis Stanikunas, Remigijus Bliumas, Karolina Jocbalyte, Algirdas Novickovas, Alvydas Soliunas

**Affiliations:** 1 54694 Institute of Psychology, Vilnius University, Lithuania; 2 Institute of Photonics and Nanotechnology, Vilnius University, Lithuania; 3 Institute of Biosciences, Vilnius University, Lithuania

**Keywords:** eye movements, phantom array, temporal processing, space constancy, flicker

## Abstract

When the eye moves through a constantly flashing light, we can observe the so-called phantom array—a line of light dashes. Our approach involves varying the flashing frequency of the light stimulus, aiming to find how the spatial and temporal characteristics of the phantom array depend on the frequency of light. The observers performed a 40° saccade across a flashing light and had to indicate the beginning and end of the phantom array, the length of a single dash, and the number of dashes they perceived. The trials of different flashing frequencies were varied randomly from 50 Hz to 3.5 kHz. We found that the number of perceived dashes was much smaller, and the perceived length of the phantom array was much shorter than the physical stimulation. However, the perceived length of dashes was the same as physically projected on the retina. Our results suggest a two-stage process which operates in phantom array perception: number compression and information processing by quantum mechanism.

## Introduction

To maintain stability of the surrounding world, the visual system must recalculate the position of each object before, during, or after saccadic eye movement. We perceive a stable image before and after a saccade but usually do not perceive image smearing during a saccade. Therefore, the image movement produced on the retina during saccade is discarded by the visual system to maintain the perceptual stability of the surrounding world. This is possible for objects with low luminance contrast. If the object luminance contrast is high, the visual system has difficulties discarding those object projections on the retina, and they appear as additional objects in the global scene perception. A stationary high-contrast object produces a light streak during a saccade (Campbell & Wurtz, 1978), and a flashing high-contrast object produces a phantom array (Hershberger, 1987). Both of those images quickly diminish from perception due to the image fading phenomenon (Martinez-Conde et al., 2004), but high-contrast objects remain longer in the visual system, and low-contrast objects with backgrounds decay more quickly (Hock & Schöner, 2023). The question is, what mechanisms govern image decay and extra image appearance during saccade? Could we explain it with a simple decay function in serial object appearances on the retina, or do higher cortex areas evaluate objects of high luminance contrast as new and put them on the perception screen as additional objects, which we perceive as illusions? In the second case, the probabilities of possible object appearances should be calculated, and a decision should be made about where to put them on the global screen of perception.

Exploring the phantom array phenomenon could shed light on the fundamental principles of visual system functioning, or more precisely, the spatial and temporal characteristics of the analysis of visual information. Under everyday conditions, this phenomenon can be observed with lightning sources that have temporal light modulation (Miller et al., 2023). The more detailed studies revealed that the phantom array is perceived approximately 2 times shorter than a physical stimulation and is mislocated in the visual field (Hershberger & Jordan, 1998; Watanabe, Maeda, et al., 2005). Watanabe, Maeda, et al. (2005) interpreted these results as indicating a compression of space during a saccade. The question arises whether the space compression in the phantom array is due to compression of separate light dashes, the distance between light dashes, or the number of dashes. Earlier, the space compression was investigated by Ross et al. (1997) and Morrone et al. (1997) in the series of experiments with very brief stimuli presentation. When four vertical bars were briefly displayed at different time intervals relative to the onset of saccades, the perceived number of bars decreased approaching saccade onset, reaching only one bar at the moment of saccade onset. Similarly, Matsumiya and Uchikawa (2001) observed a decrease in the number of bars with a four-bar stimulus. Additionally, they measured the perceived width of various stimuli and found that a single rectangle was not compressed when presented perisaccadically. Ross et al. (1997) and Matsumiya and Uchikawa (2001) suggested that space compression during saccades arises from the compression of the space between stimuli or elements of stimulus rather than from stimuli compression. The number compression was investigated by Binda et al. (2011), where they obtained a twofold decrease in perceived numerosity for a set of 30 random dots flashed perisaccadically.

The obtained spatial and temporal characteristics of the phantom array can be interpreted by a cancellation theory (Robinson, 1975; Sperry, 1950; Von Holst & Mittelstaedt, 1950), which considers two types of signals: the retinal signal of the stimulus during saccade and the internal eye position signal (EPS) (the so-called extraretinal EPS) that cancels retinal displacement caused by the eye movement. This theory basically predicts the decrease in the length of the phantom array and the mislocation of it. According to this theory, the spatial location of each individual flash is recalculated during saccade (Hershberger, 1987). Later, the author expanded his theoretical interpretation, claiming that EPS is discretely sampled 2 times during the saccade—at the saccade onset and offset—and visual coordinates (local retinal signs) are shifted at that moment (Jordan & Hershberger, 1994). However, Watanabe, Noritake, et al. (2005) reject this interpretation of cancelation theory for perisaccadic flicker perceptions and propose a two-stage localization process according to which the spatial properties of a stimulus are first established based on retinal stimulation and then the established stimulus is localized in the visual field based on eye movement signal. In other words, the data are not updated continuously and separately for each light dash during a saccade but could be determined discretely at a certain point in time (80 ms before saccade onset, according to Jordan & Hershberger, 1994). Also, perisaccadic perception of flashing stimulus could be explained by quantum perception theory (Geissler, 2018; Vanagas, 2001) that sensory information should be accumulated during a limited time interval to increase the probability of new object appearance, and only reaching some probability threshold would a new object be projected to the perception screen. This decision-making process should happen in short time intervals, which are called quantum. To investigate this quantum mechanism in greater depth, we should present objects on the retina during saccades, controlling both the duration of the stimulus and the time interval between stimuli. Our approach involves varying the flashing frequency of the light stimulus, while observers make saccades across it, aiming to find how the spatial and temporal characteristics of the phantom array depend on the frequency of light flashes. For instance, as the frequency increases, certain dashes in the phantom array might be omitted or amalgamated, resulting in a smaller number of dashes with unchanged lengths and a perceived reduction in the overall length of the phantom array.

We assume there are time intervals, or “time quanta,” during which information regarding the position and size of light dashes in the phantom array is analyzed and integrated. If this occurs during a saccade, the visual system cannot receive feedback about the true position and size of the light dashes, and errors are made. By altering the frequency of light flashes, we anticipate observing how the characteristics of the phantom array vary with the flashing frequency. Specifically, the length of the phantom array, the dash length, or the number of dashes can depend on the flashing frequency. The dependence can be a periodic or staircase function of frequency. An alternative case is that, due to the integration of space and time, both the length of the entire phantom array and the length of individual dashes would be perceived as shorter. The period or step size of the dependency should reflect the duration of the time integration process.

Hence, the aims of the experiment were as follows: first, to determine the location and length of the phantom array of flashing stimulus during saccade; second, to investigate the compression of space and number of dashes in the phantom array; and finally, to determine how these parameters depend on stimulus flashing frequency.

## Methods

### Observers

Nineteen observers with normal or corrected-to-normal visual acuity participated in the experiments. Observers’ ages ranged from 20 to 50 (average = 24.6, *SD* = 8.76). Two observers were highly experienced in performing psychophysics experiments and were familiar with the objectives of the experiments.

### Apparatus and Stimuli

**Figure 1.**
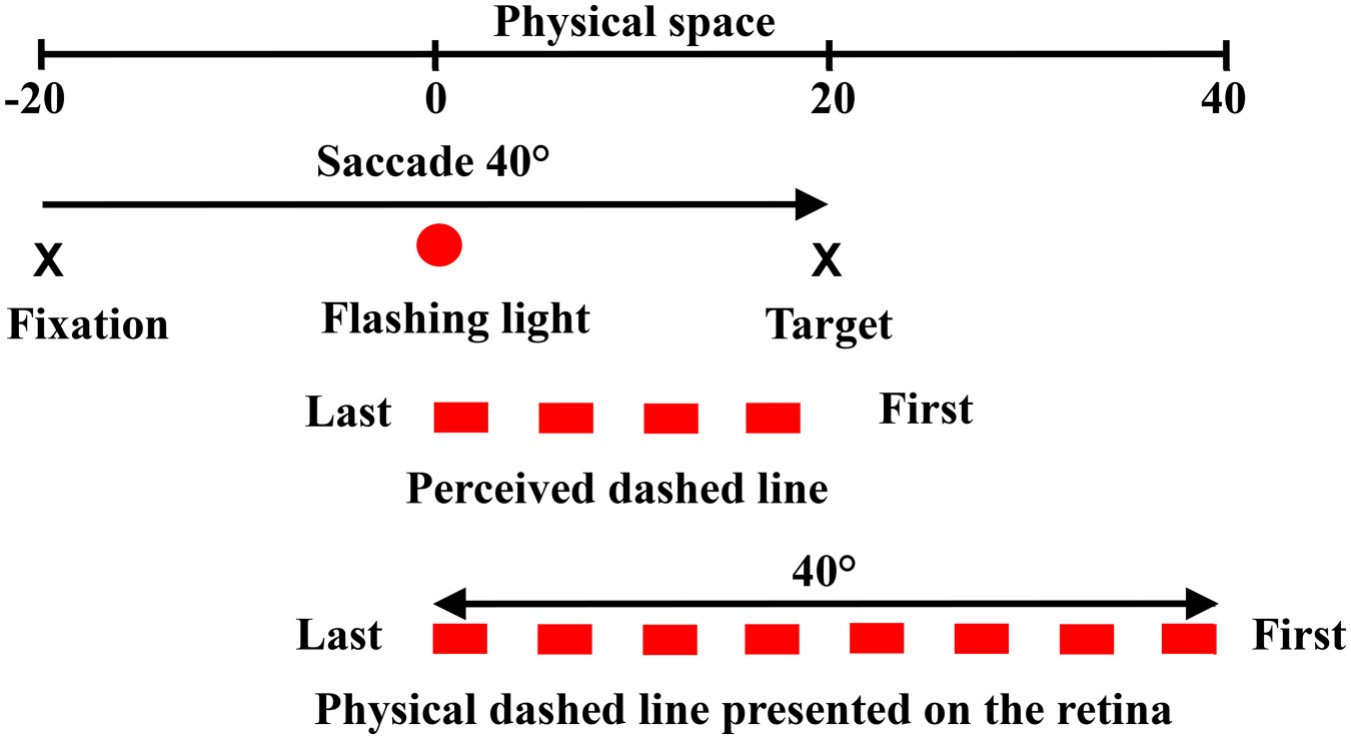
The schema of stimuli and phantom array in the space. The space scale above represents the distance in angular degrees. The presented length of perceived dashed light is just an example.

The experiments were conducted in a darkened room. Stimuli were presented in a rectangular viewing chamber with dimensions of 94 × 59 × 61 cm (W × H × D). The inside of the chamber was painted a gray color. The red light-emitting diode (LED) was put in the small box with an aperture of 0.24° diameter with diffusor, and the box was mounted in the viewing chamber at eye level. The intensity of the red light was 63 cd/m^2^, and the background was 0.03 cd/m^2^. Two additional marks serving as fixation and target points were put on the back wall of the chamber, one 20° to the left of the stimulus and another 20° to the right of the stimulus (Figure 1). To regulate the intensity and timing of the stimuli, a DAC computer board and a custom-made power driver board were employed. A computer program was used by the experimenter to control the stimulus parameters. The observer was asked to make a saccade from the fixation to the target point, while the red light stimulus was flashing in the middle of those two points. We used 50% duty cycle square wave signal for sensory stimuli with the flashing frequency varied from 50 Hz to 3.5 kHz. The distance from the observer to the stimulus was 122 cm. The observer's head was stabilized by a chin and headrest. Before experiments with flashing stimuli, the observers’ horizontal eye speed and amplitude were measured at a 200 Hz sample rate by an SMI RED250 eye tracker when the observers had to move their gaze from the fixation spot to the target spot at least 10 times. The measure of saccade speed was used to calculate the physical stimulation of the retina during eye movement. The amplitude of eye movements varied from trial to trial in the range from −20% to +0.5% from 40°. Furthermore, we conducted control experiments in which the observers shifted their gaze in a double-step saccade; first from left to center and then from center to right. The location of the phantom array immediately shows whether the saccade was a single step or double step. Not only does the length of the phantom array differ, but also the location in space. We have not recorded such discrepancies of phantom array location in our main experiment.

### Procedure

The observers adapted to the dim room for 15 min. During adaptation, the pilot trials were run, where observers were asked whether they saw something unusual when moving their eyes across a flashing light. If they saw a phantom array, they had to report the length of the phantom array and the length of one dash and count the number of dots or dashes they perceived in the phantom array. At the beginning of each trial, the observers were asked to look at the fixation point. Then the experimenter switched on the flashing light and gave the command to move the eyes (make a saccade) to the target point and fixate on the target point. After 2 s the flashing light was automatically switched off, and the experimenter asked the observers to indicate the beginning and end of the phantom array. That could be done with a laser pointer or readings from the ruler that was attached to the viewing chamber. After the answer about phantom length, the experimenter asked to report the length of one dash. That could be done with a laser pointer if the dash is long, or evaluated by the ruler. The next question was: how many dashes did the observer perceive in the phantom array? In each trial, the eye movements were repeated 3 to 15 times until the observers gave all the required answers. The trials of different flashing frequencies were randomly varied from 50 Hz to 3.5 kHz (altogether 22 predefined values of frequencies) in one experimental session. The duration of the experimental session varied from 1 to 2 hr, depending on the observer. Each observer completed three trials with each frequency in separate experimental sessions on different days.

### Data Analysis

The left and right ends of the phantom array, the number of light dashes, and the length of one dash were measured for each flash frequency. The length of the phantom array was calculated from the measures of the its beginning and end values. The data had a normal distribution according to the Shapiro–Wilk test. The effect of the flashing frequency on the length of the phantom array was estimated using a one-way analysis of variance (ANOVA) and a Fisher Least Significant Difference (LSD) post hoc test.

To report the required parameters, observers had to saccade many times in one trial. Since the particular parameter (e.g., the number of dashes) cannot be attributed to a particular saccade and consequently to a particular saccade speed, we used the averaged saccade speed for further data analysis, measured for each participant just before the experiment (see Appendices, Table A2).

## Results

### The Location and Length of the Phantom Array

**Figure 2.**
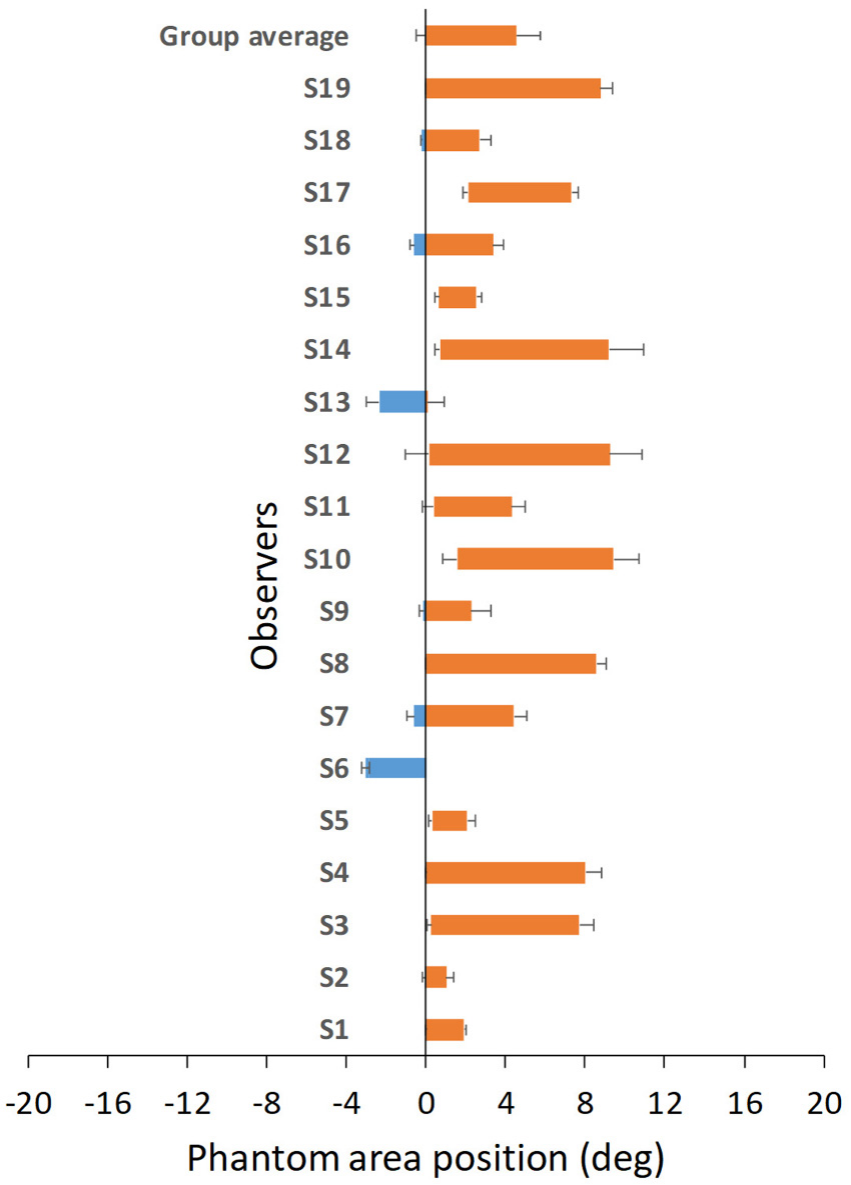
The location of the phantom array relative to the center of the visual field/saccade for different observers. Error bars represent 95% CI. Average results from all flashing frequencies are presented.

The results of the phantom array location are presented in Figure 2. The 15 observers perceived the phantom array to the right of the center. Three observers (S7, S16, and S18) perceived a phantom array on either side of the center, and the observers S6 and S13 perceived it almost exclusively to the left of the center. It should be noted that the left edge of the perceived phantom array was near or precisely in the center (“0” position), except for the mentioned observers S6 and S13.

The perceived length of the phantom array was 4.29° (±3.47 SD) on average, that is, 9.2 times shorter than the physical stimulation length. Large individual differences were obtained in phantom length estimation (significant observer effect on phantom array length in one-way ANOVA: *F*(1, 18) = 34.524, *p* < 0.0001). The shortest perceived phantom array was 0.43°, and the longest was 8.59°. For those who perceived a very short phantom array, only one or a few light dashes were visible.

**Figure 3.**
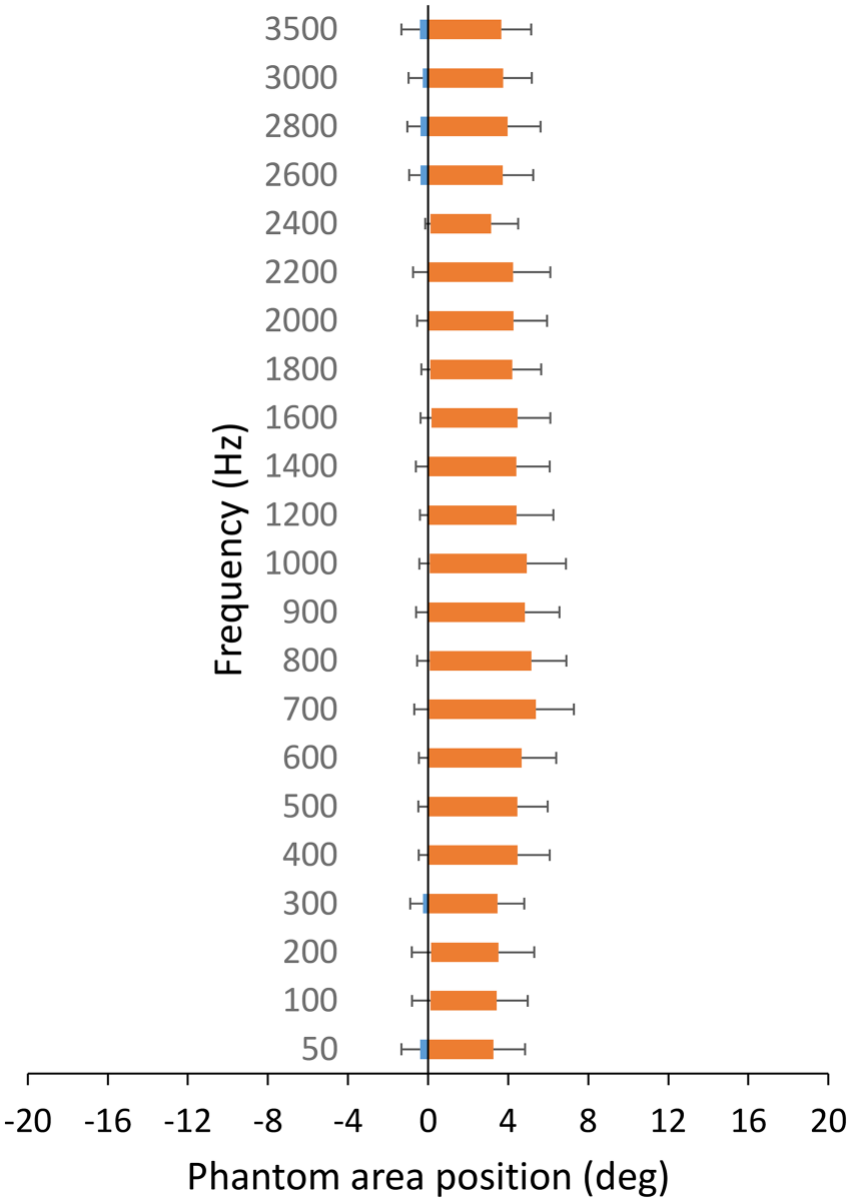
The location and length of the phantom array relative to the center of the visual field/saccade as a function of flash frequency. Average results from all observers. Error bars represent extreme left and right positions.

Also, we investigated how the length of the phantom array depends on flash frequency. These results are presented in Figure 3. A one-way ANOVA revealed a significant main effect of frequency on the perceived length of the phantom array: *F*(1, 20) = 2.380, *p* < 0.001. The Fisher post hoc test revealed that the longest phantom array was perceived at 700 Hz and 800 Hz flashing frequencies (see Table A1 in the Appendix), and the shortest at 2400 Hz flash frequency. Also, we tested the saccadic displacement effect on frequency. No effect of flashing frequency on the location of the left edge of the phantom array (Figure 3) was found, *F*(1, 20) = 0.5249, *p* = 0.956.

**Figure A1. fig7-03010066261421536:**
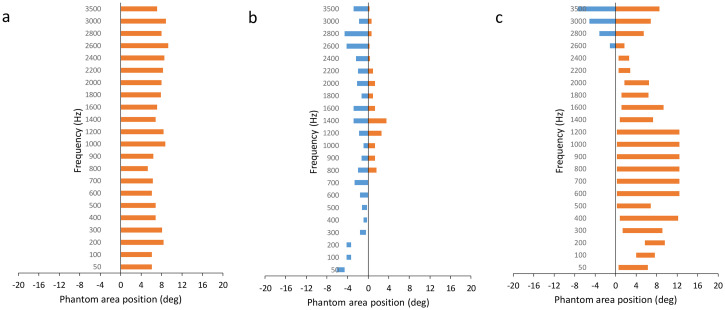
The location and length of the phantom array relative to the center of the visual field/saccade as a function of flashing frequency for. Each bar is an average result from three trials. The typical dependence for observer S8 (a) and atypical dependencies for observers S13 (b) and S12 (c).

Unfortunately, the averaged across observers’ data hide individual differences where various dependencies of phantom array length and location as a function of flash frequency were observed. Figure A1 in the Appendix demonstrates three examples of such individual differences.

### The Number and Length of Dashes in the Phantom Array

**Figure 4.**
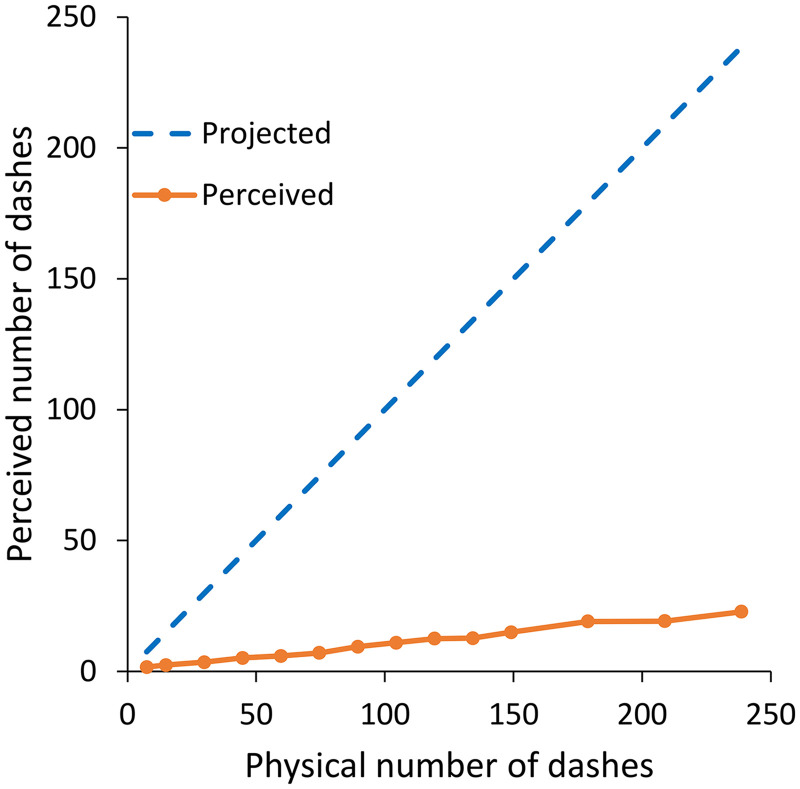
Perceived number of dashes against projected physical number of dashes in the phantom array. Dashed line shows equality.

The experimental data (solid line) vs physical stimulation data for the number of dashes are presented in Figure 4. Physical stimulation dependence was calculated for each observer according to the speed of saccades of this observer. As the perceived length of the phantom array was much shorter than the physical, the number of perceived dashes was also much smaller than the physical. Observers perceived 10.53 dashes on average, while the physical number of dashes is 103.85, that is, 9.9 times higher. Experimental and physical data discrepancy is basically the same for the phantom array length and the number of dashes in the phantom array (9.2 and 9.9 times, respectively).

**Figure 5.**
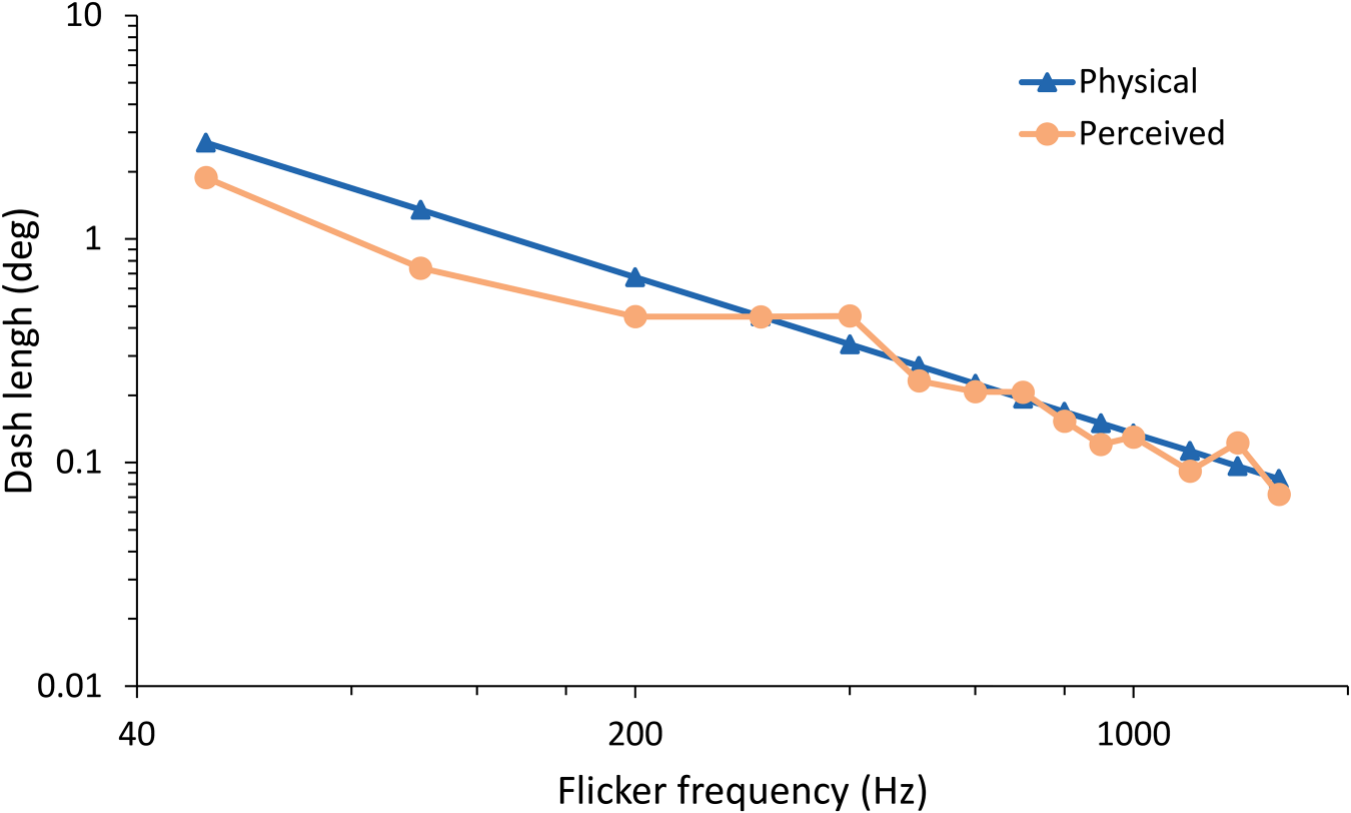
Physical and perceived length of one dash in the phantom array as a function of flash frequency.

Figure 5 presents the average length of dashes in the phantom array. The length of physical stimulation was calculated for each observer according to the speed of the saccades. We do not see the ideal coincidence of physical and experimental curves, but it could be concluded that the perceived length of dashes was reasonably close to the physical length, as should be expected based on the similar discrepancy between the physical and perceived length of the phantom array and the number of dashes in the phantom array (9.2 and 9.9 times difference, respectively). The majority of observers were able to estimate the length and number of dashes only for the 50–1600 Hz frequency range (Table 1), and therefore, Figure 5 represents only these frequencies. There was one observer (S2) who reported very short dashes for all flash frequencies, much shorter than physical for him (the lower frequency, the more mismatch between reported and physical length), one observer (S4) who reported about 3 times longer dashes than physical for him, and one observer (S11) who reported 4 or more times longer dashes than physical for him.

**Table 1.** Number of observers who reported the number of dashes for various frequencies.

Frequency (Hz)	50	100	200	300	400	500	600	700	800	900	1000
Number of observers	18	18	18	18	19	19	19	18	17	17	16
Frequency (Hz)	1200	1400	1600	1800	2000	2200	2400	2600	2800	3000	3500
Number of observers	12	11	10	3	3	3	1	1	1	1	1

## Discussion

### The Length of the Phantom Array

In our experiment, the length of the phantom array was observed to be much shorter than the length of the physical stimulation of the retina. Observers reported seeing a phantom array that was, on average, 9 times (from 4.5 to 48 for different observers) shorter than a physically stimulated area. Several studies have shown the double compression of the phantom array (Hershberger & Jordan, 1998; Watanabe, Noritake, et al., 2005). In a study similar to ours, Noritake et al. (2005) observed 40% compression of length with quite big variability ranging from 10% to 60%, depending on the observer. This compression is significantly less than our findings.

The shrinking of the phantom array can be explained by several factors. The first of these is saccadic suppression. It has been described by many authors (Matin, 1974; Volkmann, 1986, for a review). Saccadic suppression begins just before the saccade and lasts up to 50 ms after saccade onset. During saccadic suppression, the contrast threshold increases reaching a maximum at the moment of saccade initiation and then gradually decreases to a normal level. According to various authors, saccadic suppression is characteristic of the magnocellular channel for stimuli of lower spatial frequencies (Braun et al., 2017; Burr et al., 1994; Diamond et al., 2000; Gu et al., 2014; Knöll et al., 2011; Uchikawa & Sato, 1995). Assuming that saccadic suppression remained active and exerted its full effect, we estimated the length of the phantom array, excluding the first 50 ms from saccade onset, when the phantom array should not be visible. The average saccade duration was 149.1 ms, and therefore the phantom length should decrease by 33.53% (ranging between 30% and 41% across observers, depending on saccade duration, which varied between 122 and 174 ms depending on the observer) due to saccadic suppression, which is roughly one-third of its original length. However, based on the observers’ data, the observed shortening was 89.13% on average, significantly greater than anticipated. Also, we have not found a correlation between saccade speed and phantom length for different observers.

Another known phenomenon that can reduce the perceived length of the phantom array is saccadic space compression. Most authors who study visual effects during saccades and discuss spatial compression rely on studies in which the observer inaccurately estimates the spatial location of the single stimulus presented during the saccade. Ross et al. (1997) and Morrone et al. (1997) reported the significant compression of space reaching 20° at the onset of a saccade that corresponded to the amplitude of the saccade, that is, the compression reached 100%. Moreover, Matsumiya and Uchikawa (2001) investigated the compression of the shape of an object during saccades and reported that the size of an object is not compressed, but the space compression is due to the compression of space between objects or elements that are not compressed. Visual space compression was also obtained with more complex stimuli, such as natural images. Ross et al. (1997) presented an image of the Sydney Opera House, and the observers perceived it compressed about 2 times. According to Matsumiya and Uchikawa (2001), the compression of the image of Sydney Opera House does not contradict the idea that the objects are not compressed during saccades, as the complex image consists of many smaller elements and the space between these elements is compressed. In the study by Watanabe, Maeda, et al. (2005), 2D images could be generated by vertically positioned light sources flashed at different times during a saccade. The observers observed images compressed horizontally by about 2 times.

If we take into account two effects, the saccadic suppression at the onset of saccade and the space compression during saccade, they can explain the considerable shortening of the perceived phantom array in our experiment. Still, it does not explain very large individual differences and much higher shortening of the phantom array in our experiment compared to other studies of the phantom array perception.

### The Location of the Phantom Array

Based on the trace left by the flashing light on the retina, the phantom array could be visible from 0° (center) to 40° to the right if the observer made a 40° saccade as required, starting at the fixation point at −20° and stopping precisely at the target point placed at 20° (see Figure 1). However, not all saccades precisely reach the target, and the end of a saccade could deviate from the target point. We do not measure saccade amplitude in the main experiment, but we measured saccade speed and amplitude in the control experiment, where we have found no substantial variation in the saccade amplitude. Most observers saw the phantom array to the right from the center, but three observers perceived the phantom array on either side of the center, and two others perceived it almost exclusively to the left of the center (Figure 2). All this allows us to talk about the displacement of the perceived phantom array concerning physical stimulation during saccades.

Displacement of perceived visual stimuli during a saccade is a well-known phenomenon (Hershberger et al., 1998; Honda, 1993; Jordan & Hershberger, 1994; Mackey, 1970; Matin & Pearce, 1965; Ross et al., 1997). It is also called the perisaccadic shift. The popular explanation relies on neural signals sent from the oculomotor system to the visual perception system, which compensate for the displacement of the retinal image during a saccade (Sperry, 1950). These signals are called extraretinal EPSs. On the other hand, Matin and Pearce (1965) shifted the image without moving the observer's eyes and still observed the mislocation effect, suggesting that an explanation of the perisaccadic displacement relying solely on extraretinal EPSs may not be sufficient.

Most of the studies on the displacement of a perceived stimulus during a saccade have been conducted in a situation where the stimulus is presented very briefly at various time points relative to the onset of the saccade (Honda, 1993; Jordan & Hershberger, 1994; Ross et al., 1997; Watanabe, Noritake, et al., 2005). When the stimulus spatial location was in the middle between fixation and target points, Honda (1993) observed maximal stimulus mislocation toward the saccade goal when the stimulus was presented before saccade onset and maximal mislocation toward the fixation point when the stimulus was presented at the end of the saccade. However, for the same stimulus spatial location, Ross et al. (1997) observed that the perception of the stimulus shifts only in the direction of the eye movement, reaching a maximum displacement at the saccade onset. The similar mislocation was reported by Watanabe, Noritake, et al. (2005). In our experiment, the phantom is perceived between 0° and 20° for most of our observers, indicating that the spatial location of each individual flash could be recalculated during saccade (Hershberger, 1987). However, for five observers, the phantom tail was located to the left from the central point, and for two observers, all the phantom was located to the left from the central point. The similar observations were reported by Hershberger and Jordan (1998), where 15% of observers located the phantom array opposite the direction of gaze. These findings can be interpreted as an unsystematic displacement of the perceived stimulus that depends on individual observers. The obtained individual differences could perhaps be explained, according to O’Regan (1984), by the fact that the shift is caused by response differences in the visual system to central and peripheral stimuli, which may differ between observers.

According to Jordan and Hershberger (1994), the displacement of the perceived continuously flashing stimulus is determined only by the presaccadic displacement of the first visible light spot to the intended endpoint of the saccade at 80 to 0 ms before saccade onset. After seeing the first light spot (far right, if the eye moves from left to right), all other light spots appear farther and farther to the left up to the location of the blinking light. However, Watanabe, Noritake, et al. (2005) proposed a two-stage localization process according to which the spatial properties of a stimulus are first established based on retinal stimulation, and then the established stimulus is localized in the visual field based on the eye movement signal.

### The Number and Length of Dashes

We asked observers to estimate the number of dashes of light they saw in the phantom array to determine whether the dashes could be combined (or omitted) during a saccade. Indeed, a large difference was obtained between the number of dashes projected on the retina (calculated by the duration of the observer's saccade and the flash frequency) and the number of dashes seen. The observers saw them on average 9.9 times less. Perisaccadic stimulus number compression was observed in the several studies. Ross et al. (1997) and Matsumiya and Uchikawa (2001) reported that four bars presented simultaneously on the screen result in the one bar perception at the saccade onset. Binda et al. (2011) presented the number of elements in a random test array flashed at the time of a saccade and observed 35% number compression. However, in our experiment, the stimuli are presented one by one during the saccade, resulting in the phantom array perception. We discussed above the potential effects of perisaccadic suppression and spatial compression on the perception of phantom array length. Exactly the same arguments apply to the influence of these effects on the perception of the dash numbers because we found that both parameters, the length of the phantom array and the number of dashes, are directly related—the number of perceived dashes decreases by the same amount as the phantom array is shortened. In other words, the observers saw as many dashes as there should have been physically, given the length of the perceived phantom array.

In our study, the observers evaluated not only the length of the phantom array but also the length of dashes. With the reference to Figure 5, it can be inferred that the observers perceived dashes of a similar length to those projected on the retina. The same object size perception constancy during presaccadic compression of visual space was reported by Matsumiya and Uchikawa (2001), where a rectangular object was not compressed. Moreover, in our experiment after the evaluation of the length of dashes, the subjects were asked to report if the dashes in the phantom array were distributed equally and to evaluate the space length between dashes. All observers perceived equal distribution of dashes in the phantom array with space/dash ratio from 0.5 to 0.8 depending on the flash frequency. For the higher frequencies, the space between dots is perceived as shorter than the dot width, while for lower frequencies, the dash length and the space between dashes are perceived as equal. Therefore, the objects and the space between objects in the phantom array are not compressed, but we cannot rule out the possibility that the shorter perceived length of the phantom array itself could have been due to dash omission or dash combining resulting in the number compression.

### The Stimulus Flash Frequency

We cannot compare the dependence of the perceptual characteristics of the phantom array on the flashing frequency obtained in our experiment (Figure 3) with other similar studies where researchers (Hershberger, 1987; Hershberger et al., 1998; Hershberger & Jordan, 1998; Jordan & Hershberger, 1994; Watanabe, Maeda, et al., 2005) investigated phantom array perception in psychophysical experiments similar to our investigation, as they used only one flashing frequency (120, 200 , 250, or 500 Hz, depending on the study). On the other hand, there are a number of studies in the temporal light modulation area where observers evaluated phantom array visibility, that is, whether they see the phantom array at different stimulus frequencies (Park et al., 2020; Wang et al., 2020; Yu et al., 2018). In such studies, the best visibility (the lowest threshold) of the phantom array was found at a stimulus flashing frequency of about 600 Hz. Assuming that in our study the threshold for the visibility of the phantom region is also lower at the 600 Hz flash frequency, we would expect a longer phantom length at this frequency. Indeed, observers reported a longer (5.0°–5.3°, as compared to about 4°) phantom array when the stimulus flashed at 700 and 800 Hz. The phantom array was, on average, ¼ longer at these frequencies compared to other frequencies (Figure 3). However, we found large variations in phantom position and length between observers. Therefore, according to suggestions by Peterzell and Kennedy (2016), we analyze individual data for observers (see Figure A1 in the Appendix). Seven observers demonstrated a typical dependence similar to the dependence for observer S8 (Figure A1a in the Appendix) that is, the position was to the right side of the flashing stimulus, and the length remained more or less stable for various flashing frequencies. For four observers, the phantom array position was shifted to the left when the flashing frequency decreased or increased (Figure A1b in the Appendix). For observer S12, the phantom array shifted to the left when the flashing frequency increased, and it shifted to the right when the flashing frequency decreased (Figure A1c in the Appendix). For four observers, the location of the phantom array varied unsystematically with the changing frequency of the flashing stimulus.

### Quantum Mechanism of Phantom Perception

According to Geisler's Time Quantum Model (Geissler, 1990; 2018; Geissler et al., 2012; Geissler & Reschke, 1987), all perceptual (and not only perceptual) processes in the brain go discretely, and the smallest time interval, called the time quantum (*TQ*), is 4.57 ± 0.04 ms (Geissler et al., 1999). The actual operation times of processes in the brain are multiples of *TQ*, that is, *N* *×* *TQ*, where *N* ranges from 1 to 30. Having the responses of observers about the length of the phantom array, the perceived number of phantom dashes, and knowing the number of physical dashes, we can calculate the integration time (*Q*) during which the phantom dashes are accumulated into one percept:

$$Q=(T_{s}\text{–}\;50)\times n_{p}/n_{\;ph},$$

where *T_s_* is the saccade duration in milliseconds; *n_p_* is the number of perceived dashes; *n_ph_* is the number of physical dashes; and 50 is the 50-ms duration of saccadic suppression.

**Figure 6.**
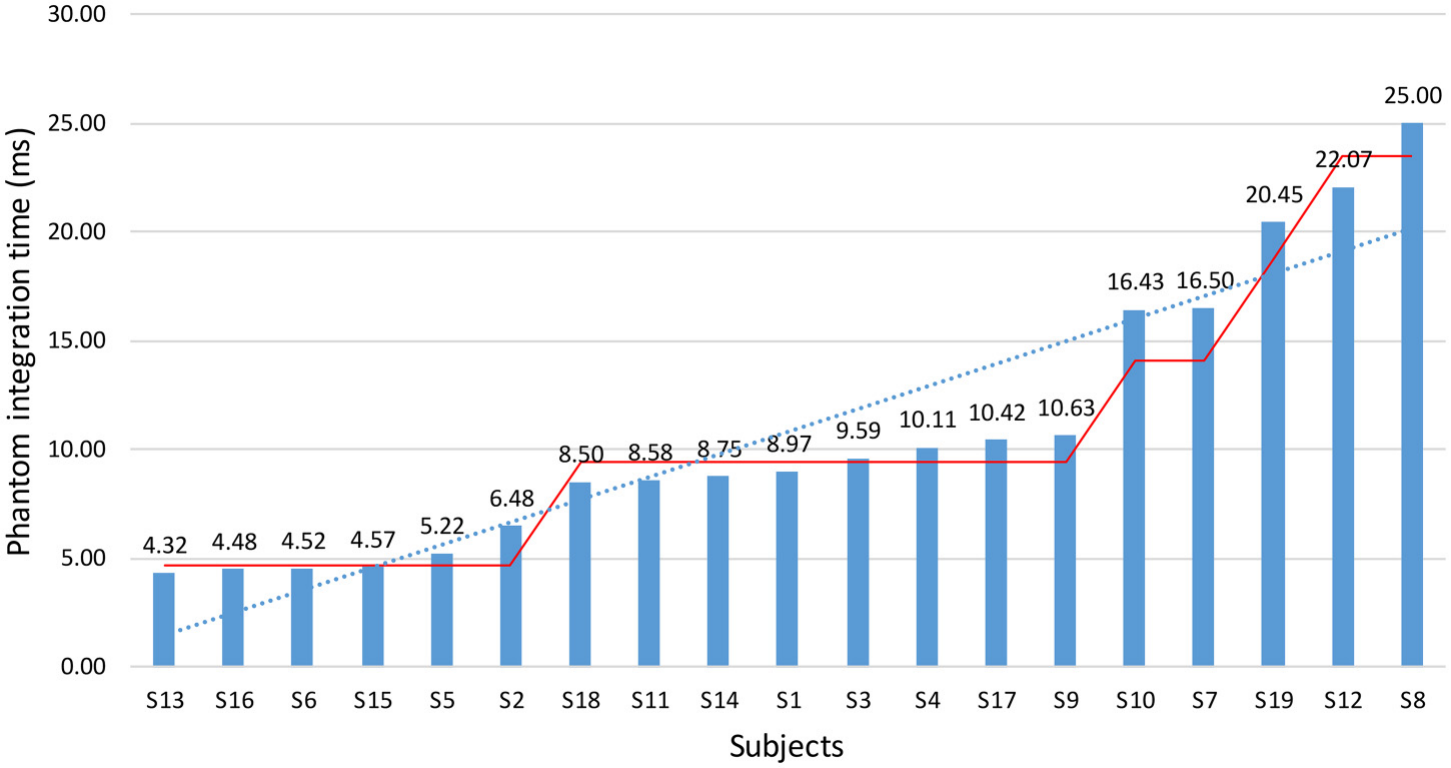
Integration time of phantom perception for different observers. The solid curve represents the theoretical curve corresponding to the quantum model. The dotted curve represents the linear regression of the data.

The obtained integration time values are presented in Figure 6. The values are presented in increasing order. We can see that the values are not random or normally distributed but could be arranged in a step-like function (*R*^2^ = 0.968). The smallest average value for five observers is 4.62 ms, and the next value is 9.44 ms (for eight observers), which is about 2 times bigger. For comparison, the *R*^2^ for the linear regression curve with experimental data is 0.796. Surprisingly, our data are consistent with Geisler's 4.57 ms *TQ*. If we accept Geisler's theory, we can speculate that six observers (S2, S5, S6, S13, S15, S16) integrate information about flashing light during one quantum and perceive as many dashes as can be projected on the retina during one quantum. Observers S1, S3, S4, S9, S11, S14, S17, and S18 perceive a phantom array that was integrated during two quanta. Accordingly, other observers’ perception operates in two, four, or five quanta.

## Conclusion

Our results revealed that all observers perceived the light dash length in the phantom array to be the same as the physical projection on the retina. Despite the substantial interobserver variability, the perceived length of the phantom array was much shorter than the physical stimulation array. Similarly, the number of light dashes in the phantom array was much smaller than the number of light stimuli presented on the retina. Our results suggest a two-stage process that operates in phantom array perception: number compression and information processing by quantum mechanism.

## Appendix

[Table table2-03010066261421536] [Table table3-03010066261421536] [Fig fig7-03010066261421536]

**Table A1. table2-03010066261421536:** Fisher LSD post hoc test results of differences in phantom array length for different frequencies. Significant cells are indicated thus: **p* < .05.

Frequency (Hz)	50	100	200	300	400	500	600	700	800	900	1000	1200	1400	1600	1800	2000	2200	2400	2600	2800	3000
50		0.2808	0.1073	0.6460	0.8036	0.4913	0.3823	0.0262*	0.0610	0.1333	0.3544	0.9204	0.6285	0.8347	0.5574	0.8626	0.7279	0.0234*	0.6737	0.5405	0.3331
100	0.2808		0.5934	0.5353	0.1847	0.0777	0.0514	0.0010*	0.0033*	0.0101*	0.0455*	0.2387	0.1221	0.4105	0.6328	0.3813	0.4940	0.2146	0.5413	0.6745	0.9492
200	0.1073	0.5934		0.2489	0.0632	0.0218*	0.0133*	0.0001*	0.0005*	0.0020*	0.0115*	0.0873	0.0385*	0.1827	0.3154	0.1636	0.2326	0.4645	0.2624	0.3524	0.5659
300	0.6460	0.5353	0.2489		0.4790	0.2514	0.1829	0.0074*	0.0199*	0.0503	0.1663	0.5761	0.3491	0.8177	0.8936	0.7846	0.9272	0.0671	0.9855	0.8627	0.5967
400	0.8036	0.1847	0.0632	0.4790		0.6601	0.5318	0.0481*	0.1038	0.2100	0.4980	0.8817	0.8115	0.6552	0.4055	0.6783	0.5581	0.0124*	0.5101	0.3956	0.2282
500	0.4913	0.0777	0.0218*	0.2514	0.6601		0.8527	0.1236	0.2346	0.4152	0.8119	0.5562	0.8452	0.3861	0.2060	0.3999	0.3146	0.0036*	0.2808	0.2044	0.1044
600	0.3823	0.0514	0.0133*	0.1829	0.5318	0.8527		0.1754	0.3156	0.5291	0.9582	0.4389	0.7051	0.2966	0.1479	0.3068	0.2369	0.0020*	0.2093	0.1482	0.0718
700	0.0262*	0.0010*	0.0001*	0.0074*	0.0481*	0.1236	0.1754		0.7245	0.4673	0.1927	0.0337*	0.0867	0.0197*	0.0056*	0.0197*	0.0135*	0.0001*	0.0110*	0.0063*	0.0021*
800	0.0610	0.0033*	0.0005*	0.0199*	0.1038	0.2346	0.3156	0.7245		0.7079	0.3415	0.0760	0.1716	0.0456*	0.0151*	0.0463*	0.0326*	0.0001*	0.0271*	0.0165*	0.0060*
900	0.1333	0.0101*	0.0020*	0.0503	0.2100	0.4152	0.5291	0.4673	0.7079		0.5640	0.1610	0.3180	0.1003	0.0390*	0.1028	0.0748	0.0002*	0.0637	0.0410*	0.0165*
1000	0.3544	0.0455*	0.0115*	0.1663	0.4980	0.8119	0.9582	0.1927	0.3415	0.5640		0.4086	0.6672	0.2740	0.1340	0.2834	0.2177	0.0017*	0.1917	0.1347	0.0643
1200	0.9204	0.2387	0.0873	0.5761	0.8817	0.5562	0.4389	0.0337*	0.0760	0.1610	0.4086		0.7000	0.7610	0.4932	0.7870	0.6575	0.0182*	0.6055	0.4793	0.2877
1400	0.6285	0.1221	0.0385*	0.3491	0.8115	0.8452	0.7051	0.0867	0.1716	0.3180	0.6672	0.7000		0.5034	0.2912	0.5213	0.4200	0.0070*	0.3796	0.2860	0.1563
1600	0.8347	0.4105	0.1827	0.8177	0.6552	0.3861	0.2966	0.0197*	0.0456*	0.1003	0.2740	0.7610	0.5034		0.7221	0.9701	0.8937	0.0480*	0.8385	0.6987	0.4661
1800	0.5574	0.6328	0.3154	0.8936	0.4055	0.2060	0.1479	0.0056*	0.0151*	0.0390*	0.1340	0.4932	0.2911	0.7221		0.6893	0.8273	0.09278	0.8839	0.9659	0.6932
2000	0.8626	0.3813	0.1636	0.7846	0.6783	0.3999	0.3068	0.0197*	0.0463*	0.1028	0.2834	0.7870	0.5213	0.9701	0.6893		0.8626	0.0410*	0.8070	0.6669	0.4370
2200	0.7279	0.4940	0.2326	0.9272	0.5581	0.3146	0.2369	0.0135*	0.0326*	0.0748	0.2177	0.6575	0.4200	0.8937	0.8273	0.8626		0.0651	0.9440	0.7998	0.5516
2400	0.0234*	0.2146	0.4645	0.0671*	0.0124*	0.0036*	0.0020*	0.0001*	0.0001*	0.0002*	0.0017*	0.0182*	0.0070*	0.0480*	0.0928	0.0410*	0.0651		0.0759	0.1113	0.2106
2600	0.6737	0.5413	0.2624	0.9855	0.5101	0.2808	0.2093	0.0110*	0.0271*	0.0637	0.1917	0.6055	0.3796	0.8385	0.8839	0.8070	0.9440	0.0759		0.8545	0.5994
2800	0.5405	0.6745	0.3524	0.8627	0.3956	0.2044	0.1482	0.0063*	0.0165*	0.0410*	0.1347	0.4793	0.2860	0.6987	0.9659	0.6669	0.7998	0.1113	0.8545		0.7324
3000	0.3331	0.9 492	0.5659	0.5967	0.2282	0.1044	0.0718	0.0021*	0.0060*	0.0165*	0.0643	0.2877	0.1563	0.4661	0.6932	0.4370	0.5516	0.2105	0.5994	0.7324	

**Table A2. table3-03010066261421536:** Saccade duration in milliseconds for observers.

Subject	S1	S2	S3	S4	S5	S6	S7	S8	S9	S10
Saccade duration (ms)	174.3	137.3	122.2	139.1	135.1	143.4	160.3	152.7	146.9	148.2
Subject	S11	S12	S13	S14	S15	S16	S17	S18	S19	
Saccade duration (ms)	170.5	140.5	170.1	137.5	131.4	154.9	166.7	163.4	138.7	
